# Risk Factors and Brain Substrates of Motoric Cognitive Risk Syndrome in Rural and Urban Residents: Results from the Kerala‐Einstein Study

**DOI:** 10.1002/alz.092427

**Published:** 2025-01-09

**Authors:** Ayers Emmeline, Helena M Blumen, Natalie A Delpratt, Dristi Adhikari, Joe Verghese

**Affiliations:** ^1^ Department of neurology, Division of Cognitive and Motor Aging, Albert Einstein College of Medicine, Bronx, NY USA; ^2^ Albert Einstein College of Medicine, Bronx, NY USA; ^3^ Department of Neurology, Division of Cognitive and Motor Aging, Albert Einstein College of Medicine, New York, NY USA

## Abstract

**Background:**

Rural populations are under‐represented in cognitive studies worldwide. The few extant studies report differences in risk profiles for pre‐dementia and dementia syndromes among urban and rural residents. We compared risk factors and brain substrates of Motoric Cognitive Risk (MCR) syndrome, a pre‐dementia syndrome identified by slow gait speed and subjective cognitive concerns, in urban and rural participants in the Kerala‐Einstein study.

**Method:**

Participants (aged 60+) are recruited from rural and urban sites in Kozhikode district, Kerala, India. Participants with possible dementia were excluded (score ≤4 on the Picture‐Memory Impairment Screen). Logistic regression was used to identify risk factors of MCR in rural and urban residents. Multivariate general linear models were used to examine the relationship between MCR and brain structures as a function of residency.

**Result:**

334 rural (*M* age = 68.7±5.1; 64% male, 20% high school graduates) and 299 urban *(M* age = 68.7±6.1; 60% male, 25% high school graduates) participants without dementia were examined. MCR prevalence was similar in rural (10%) and urban (9%) settings. Individuals with MCR in both samples reported more depressive symptoms and fewer years of education. Urban MCR participants were more likely to use a hearing aid, have apathy, and have occupations that involved manual labor. Modeled simultaneously, hearing aids (p = 0.05) and manual labor (p = 0.027) were associated with MCR in urban settings, and education years (p = 0.010) in the rural setting. In a subset of participants with MRI (*N* = 213; 145 Urban; 68 rural; 15% MCR), significant interactions between MCR and residency were observed for the right frontal cortical thickness (p = 0.005) and left hippocampal volume (p = 0.018) such that a significant association was only observed in the urban residents – after adjusting for age, sex, education, overall white matter lesion burden, total intracranial volume, and depressive symptoms. After adjusting for apathy, only the association between MCR and left hippocampal volume remained significant.

**Conclusion:**

The risk factors and the brain substrates associated with MCR differ in urban and rural residents from Kerala, India, and have implications for designing interventions and understanding the pathophysiology of MCR.

